# From Fecal Microbial Potential to Circulating Trimethylamine N-oxide and Cardiovascular Events: A Narrative Review

**DOI:** 10.7759/cureus.115807

**Published:** 2026-09-05

**Authors:** Ruxandra Florentina Ionescu, Sanda Maria Cretoiu

**Affiliations:** 1 Department of Cardiology I, "Dr. Carol Davila" Central Military Emergency University Hospital, Bucharest, ROU; 2 Department of Morphological Sciences, Carol Davila University of Medicine and Pharmacy, Bucharest, ROU

**Keywords:** cardiovascular disease, causal inference, fecal metabolome, fmo3, gut microbiota, renal clearance, trimethylamine, trimethylamine n-oxide

## Abstract

Trimethylamine N-oxide (TMAO) is frequently presented as a gut microbiota-derived cardiovascular biomarker, but this label compresses several biologically distinct transitions into a single causal narrative. Microbial gene carriage does not establish active trimethylamine (TMA) flux; a fecal TMA or TMAO signal does not quantify the absorbed dose; circulating TMAO integrates microbial and dietary input with intestinal absorption, hepatic flavin-containing monooxygenase 3 (FMO3) activity, tissue distribution, sampling time, and renal clearance; and a prognostic association does not by itself demonstrate causal cardiovascular mediation. This critical narrative review examines the limits of inference across these levels. Meta-analyses of prospective cohorts have generally reported modest associations between higher circulating TMAO and adverse cardiovascular outcomes, while recent community cohorts provide additional positive evidence. Nevertheless, effect sizes and interpretation vary across populations, and null or context-dependent findings remain important. The closest prior compartment-focused systematic review evaluated the effect of red meat on circulating, urinary, and fecal TMAO in randomized trials. The present review addresses a different and broader question: when is it valid to move from stool-based microbial or metabolite measurements to systemic exposure and cardiovascular claims? We organize the evidence as a series of gates linking microbial capacity, realized luminal metabolism, absorption, FMO3-dependent oxidation, circulating concentration, and clinical outcome. The fecal TMAO literature remains sparse, and stool microbial profiles have not reliably predicted diet-induced systemic TMAO responses. Mendelian randomization and kidney-function studies further challenge a simple unidirectional causal interpretation. We propose a matrix-specific conceptual framework for reporting and study design. At present, fecal TMAO is best interpreted as an exploratory intestinal measurement, not as a surrogate for circulating TMAO or an independently validated cardiovascular risk marker. Future studies should pair biological matrices, quantify TMA and TMAO, standardize diet and timing, characterize renal and FMO3-relevant host factors, and test mediation rather than association alone.

## Introduction and background

Trimethylamine N-oxide (TMAO) has become a prominent candidate biomarker at the intersection of diet, gut microbial metabolism, host biotransformation, and cardiovascular disease. The canonical pathway is biologically plausible and experimentally tractable: intestinal microorganisms convert nutrients containing a trimethylamine (TMA) moiety, including choline, phosphatidylcholine, L-carnitine, and betaine, into TMA; absorbed TMA reaches the liver and is oxidized predominantly by flavin-containing monooxygenase 3 (FMO3); and TMAO enters the systemic circulation and is largely eliminated through the kidneys [1-4]. Experimental studies have linked this pathway to altered cholesterol handling, atherosclerotic lesion development, platelet hyperreactivity, and thrombosis [1,3,5,6].

Prospective cohorts and meta-analyses have repeatedly associated higher circulating TMAO concentrations with major adverse cardiovascular events and mortality [7-9]. A frequently cited meta-analysis of prospective cohorts reported a 23% higher risk of cardiovascular events among participants with higher circulating TMAO concentrations (hazard ratio 1.23; 95% confidence interval, 1.07-1.42) [9]. Yet the apparent consistency of the pooled signal should not be confused with uniform certainty about its origin or causal meaning. An umbrella review published in 2025 identified 27 systematic reviews and meta-analyses, but judged confidence in the secondary evidence to be critically low because many primary studies enrolled patients with multiple comorbidities, incompletely addressed confounding, and used heterogeneous analytical and clinical definitions [10].

The human evidence is neither uniformly positive nor uniformly negative. In the Multiethnic Study of Atherosclerosis, higher plasma TMAO was associated with incident atherosclerotic cardiovascular events among 6,767 adults initially free of clinical cardiovascular disease [11]. Serial measurements in the Cardiovascular Health Study and the Multiethnic Study of Atherosclerosis were also associated with incident ischemic stroke, with an approximately 11% higher risk per doubling of plasma TMAO [12]. Conversely, circulating TMAO did not predict 10-year survival in a coronary and community comparison, and a study spanning healthy weight, overweight, obesity, and metabolic syndrome found no significant between-group differences or clear cardiometabolic associations [13,14]. These apparently discordant findings are not mutually exclusive; they indicate that TMAO performance depends on population, disease stage, renal function, diet, timing, and analytical context.

The deeper interpretive problem appears when the phrase "microbiota-derived TMAO" is used as though one continuously observed process had been measured. Cardiovascular microbiome studies may quantify fecal taxa, TMA-producing genes, predicted pathways, fecal TMA or TMAO, plasma or serum TMAO, urinary excretion, or clinical outcomes. These are related domains, but they are not interchangeable readouts. Treating a fecal or metagenomic signal as direct evidence of systemic exposure is a category error because several unmeasured biological filters lie between the stool sample and the cardiovascular endpoint [15-20].

The closest prior synthesis is the 2025 systematic review by Jafari et al., which explicitly compared circulating, urinary, and fecal TMAO across 15 publications from 13 randomized controlled trials involving 553 participants [15]. That review asked a focused empirical question: how does higher vs. lower red meat intake alter TMAO in different biological compartments? It found inconsistent short-term effects and substantially fewer fecal than circulating or urinary datasets. The present review asks a different question. It examines the validity of inference when a measurement at one biological level is used to support a claim at another, extending beyond dietary intervention to microbial gene carriage, realized TMA flux, absorption, FMO3-dependent oxidation, renal handling, Mendelian randomization (MR), and clinical mediation.

Other recent reviews have assessed TMAO as a general cardiovascular biomarker or have discussed it alongside short-chain fatty acids, bile acids, and other pathways within the broader gut-heart axis [16,17]. They are informative summaries of the field, but the fecal-vs.-circulating inferential break is not their organizing problem. Prior publications from our group have likewise considered the wider relationships among the microbiome, atherosclerosis, vascular calcification, microRNA signaling, and intestinal microbiomics [18-20]. The present article is deliberately narrower: its contribution is an evidence-gated framework for determining what each TMA/TMAO measurement can and cannot establish.

The central proposition is therefore not that circulating TMAO lacks prognostic value nor that the TMA-TMAO pathway is biologically irrelevant. It is that five propositions must remain separate: microbial potential is not realized metabolic flux; a fecal signal is not an absorbed dose; absorbed TMA does not imply a fixed FMO3-dependent TMAO yield; circulating concentration is not production rate; and epidemiological association is not causal cardiovascular mediation. The review follows these transitions stage by stage and translates them into practical reporting standards for cardiovascular microbiome research [15-20].

## Review

Structured literature search and analytical approach

This critical narrative review was informed by a structured PubMed search from database inception through July 31, 2026. The search combined the concepts of TMA/TMAO, biological matrix, microbial function, host handling, and cardiovascular outcome. The complete search expression was: (("trimethylamine N-oxide"[Title/Abstract] OR TMAO[Title/Abstract] OR trimethylamine[Title/Abstract]) AND (fecal[Title/Abstract] OR faecal[Title/Abstract] OR stool[Title/Abstract] OR plasma[Title/Abstract] OR serum[Title/Abstract] OR urine[Title/Abstract] OR FMO3[Title/Abstract] OR metagenom*[Title/Abstract] OR cutC[Title/Abstract] OR cutD[Title/Abstract] OR cntA[Title/Abstract] OR cntB[Title/Abstract] OR "renal function"[Title/Abstract] OR "cardiovascular disease"[Title/Abstract] OR atheroscleros*[Title/Abstract] OR "major adverse cardiovascular event"[Title/Abstract] OR "Mendelian randomization"[Title/Abstract])).

We included English-language primary human studies that addressed at least one transition in the proposed pathway, including prospective cardiovascular cohorts, dietary challenges and controlled feeding studies, paired fecal and systemic measurements, direct fecal metabolite studies, and investigations of FMO3 or renal handling. Foundational in vivo or in vitro studies were retained when they directly tested a relevant mechanistic transition. Systematic, umbrella, and narrative reviews were used to define the context and to identify additional primary studies. We excluded duplicate reports, conference abstracts without sufficient methodological information, articles without a direct TMA/TMAO focus, and studies that did not contribute evidence to at least one biological matrix or inferential transition.

One author (R.F.I.) conducted the search and initial study selection; both authors reviewed the final evidence set and resolved questions of inclusion by discussion. The updated synthesis included 49 publications. The number of initially retrieved records was not prospectively logged because the review was not designed as a systematic review. No formal risk-of-bias instrument or quantitative synthesis was applied. Instead, study design, biological matrix, sampling conditions, renal adjustment, and consistency across adjacent evidence levels were considered qualitatively. These choices, together with the absence of duplicate screening, are acknowledged as limitations.

The cardiovascular signal: prognostic association, heterogeneity, and confidence

The strongest case for TMAO as a cardiovascular biomarker arises from prospective plasma or serum studies. Early work demonstrated graded associations between fasting plasma TMAO and incident events after adjustment for conventional risk factors [2]. Subsequent meta-analyses pooled positive findings across coronary disease, heart failure, diabetes, chronic kidney disease, and mixed-risk populations [7-9]. More recent community-based cohorts reduce the concern that the signal is confined to secondary-prevention samples [11,12]. Accordingly, it would be inaccurate to dismiss circulating TMAO as an uninformative biomarker.

However, an adjusted hazard ratio is a population-level association, not a direct assay of microbial activity or causal mechanism. The component studies differ in fasting status, baseline kidney function, assay platform, exposure categorization, follow-up, medication burden, outcome definitions, and covariate adjustment. "Major adverse cardiovascular events" may include different combinations of myocardial infarction, stroke, revascularization, heart failure, and death. A high-vs.-low comparison may also correspond to very different absolute concentrations. Pooling can establish an average prognostic relationship while obscuring which biological process generated that relationship in a particular cohort [7-14].

The null and context-dependent studies are therefore scientifically important rather than inconvenient exceptions [13,14]. They suggest that circulating TMAO may be more informative in selected high-risk settings than in early metabolic phenotypes or relatively healthy populations. They also raise the possibility that the biomarker integrates disease burden, renal reserve, dietary exposure, and medication effects. The relevant question is not whether a statistically significant association can be found, but what interpretation is justified by the biological matrix, study design, and covariates actually measured.

The umbrella review reinforces this distinction [10]. Most secondary syntheses reported positive associations, but confidence was limited by the quality and heterogeneity of the underlying studies. Thus, the literature supports a cautious dual conclusion: circulating TMAO has reproducible prognostic potential in several populations, while the origin and causal meaning of a given concentration remain unresolved unless the upstream and downstream links are directly evaluated.

Clinical heterogeneity becomes clearer when outcome definitions and sampling settings are separated. A measurement obtained in a stable community cohort does not answer the same clinical question as a measurement obtained during acute coronary syndrome, chronic heart failure, or chronic kidney disease. The former is closer to long-term risk prediction, whereas the latter may also reflect acute hemodynamic stress, renal congestion, inflammation, medication exposure, or catabolic state. Even in prospective studies, a single baseline sample assumes a relatively stable exposure although TMAO can change with diet, kidney function, and clinical status [7-14].

A universal clinical decision threshold has not been validated across populations or biological matrices. Most studies model circulating TMAO continuously or compare study-specific quantiles, so the meaning of "high TMAO" depends on the cohort, assay, and sampling conditions. A statistically independent association is also not equivalent to useful clinical classification. Translation to practice would require external calibration, discrimination analyses, assessment of added predictive value beyond established risk models, and evidence that using the marker improves management or outcomes [7-14].

Evidence gate 1: microbial potential vs. realized TMA flux

The first inferential leap occurs when fecal taxonomic or genomic information is treated as evidence of active metabolite production. Microorganisms can generate TMA from choline, carnitine, betaine, gamma-butyrobetaine, and related substrates through several pathways. Genes such as cutC/cutD and cntA/cntB identify potential biochemical capacity, and potential TMA-producing organisms are widely distributed in mammalian microbiomes [21]. Yet gene carriage does not establish transcription, enzyme abundance, substrate access, pathway direction, or quantitative flux.

Realized TMA production is conditioned by ecological and dietary context. Substrate availability depends on recent and habitual diet, digestion, food matrix, intestinal transit, and competition among microorganisms. Gene expression and enzyme activity depend on local redox conditions and community interactions. Cross-feeding can either increase precursor availability or divert metabolites into alternative pathways. Consequently, two communities with similar gene abundance can produce different TMA outputs, while different taxonomic configurations may converge on a similar functional phenotype [21-25].

Functional redundancy further weakens simple taxon-based inference. The same methylamine-producing function can be distributed across phylogenetically distinct organisms, while members of the same genus or species may differ in relevant gene content. Relative abundance adds another limitation because it does not provide absolute microbial load and may change when unrelated community members expand or contract. A taxonomic association should therefore be interpreted as an ecological correlate, not as a quantitative assay of TMA production [21].

Producer phenotypes are also dynamic. Precursor challenges show that fasting concentrations may fail to identify individuals who generate large postexposure responses, and the response can differ according to habitual diet and microbial adaptation. Human studies combining dietary exposure, stool profiles, and systemic TMAO have found only partial correspondence among these domains. These observations support standardized challenge designs and repeated sampling when the research question concerns pathway activity rather than microbial potential alone [22-25].

Human challenge studies illustrate why a functional phenotype cannot be inferred from a static stool profile. A carnitine challenge identified high- and low-producer phenotypes that were not adequately characterized by a single fasting measure or taxonomic profile [22]. Ferrell et al. found that fecal microbiome composition did not reliably predict diet-induced systemic TMAO production in healthy adults [23]. In metabolically healthy adults, associations among diet, fecal microbial features, and plasma TMAO were modest and incomplete [24]. A small study linking urinary TMAO with fecal taxa and cutC subtypes was useful for method development but was not a validated predictive model [25].

These findings do not make microbial genes irrelevant. They define the level of conclusion those genes can support. Detection of cutC or cntA can justify a statement about potential capacity and can motivate a challenge or flux study. It cannot, by itself, establish that substantial TMA was produced, absorbed, converted to TMAO, or associated with cardiovascular risk in the same person. The evidence required to cross this first gate is functional: targeted metabolites, expression or protein activity, isotope tracing, or a standardized precursor challenge [21-25]. The proposed matrix-specific conceptual framework and the evidence required for moving between biological levels are summarized in Figure 1.

**Figure 1:**
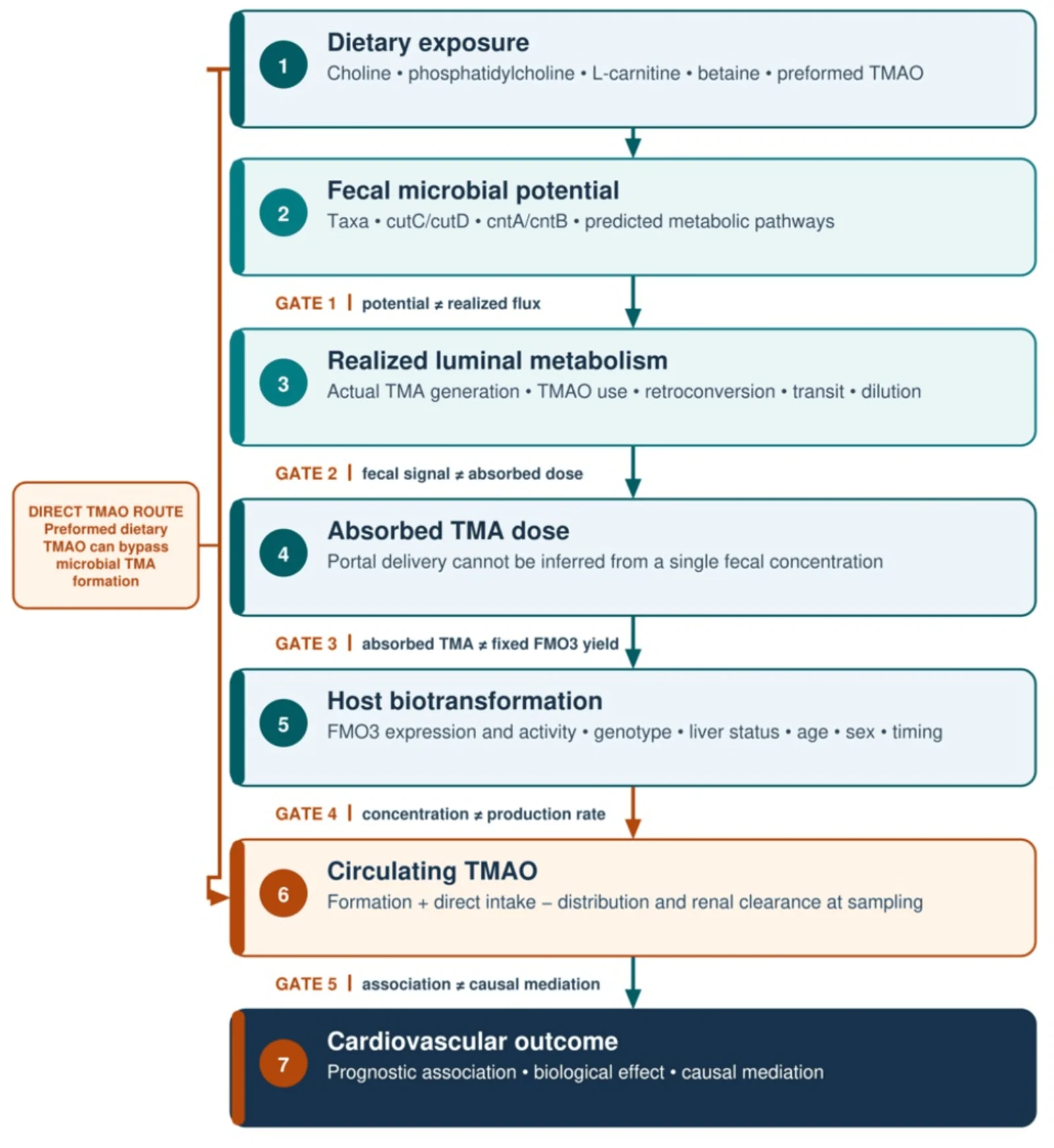
A matrix-specific conceptual framework for evidence-gated interpretation of the pathway linking fecal microbial potential, trimethylamine generation, circulating trimethylamine N-oxide, and cardiovascular outcomes The diagram illustrates sequential inferential levels from microbial potential in stool to cardiovascular outcomes, highlighting the major evidence gates that separate potential capacity, realized luminal metabolism, intestinal absorption, host biotransformation, circulating concentration, and clinical association. Measures obtained at adjacent biological levels are related but not interchangeable [2,7-15,21-25] TMA: trimethylamine; TMAO: trimethylamine N-oxide; FMO3: flavin-containing monooxygenase 3 Image credit: This figure was created by the authors using Microsoft PowerPoint (Microsoft Corporation, Redmond, WA)

Evidence gate 2: fecal signal vs. absorbed dose

The second inferential leap is the assumption that TMA or TMAO measured in stool directly represents the dose delivered to the host. Stool is a residual and compositionally variable matrix. A high fecal concentration may reflect increased luminal production, direct dietary delivery, reduced mucosal absorption, reduced microbial use, slow transit, or lower stool water content. A low concentration may reflect low production, efficient absorption, active degradation, rapid transit, or dilution. Without simultaneous information on intake, stool output, transit, and paired systemic measurements, these explanations cannot be distinguished [15,23-26].

A concentration in stool is not a mass-balance estimate. Without total stool output, a value normalized per gram cannot distinguish greater whole-gut excretion from a more concentrated specimen. Nor does it identify the fraction produced proximally and absorbed before the remaining material reached the sampled stool. Paired fecal, plasma, and urinary measurements collected over a defined interval are therefore more informative than cross-sectional comparison of isolated concentrations [15,26].

Preanalytical variation can be large relative to the biological signal of interest. Delayed stabilization, microbial activity after collection, water-content variation, freeze-thaw exposure, and differences between wet- and dry-weight normalization can alter the apparent fecal metabolite profile. Targeted assays should demonstrate recovery, linearity, precision, matrix effects, and analyte stability in stool rather than assume that validation in plasma or urine transfers automatically to the fecal matrix [15,25,26].

The intestinal pathway is also bidirectional. Hoyles et al. demonstrated microbial retroconversion of TMAO to TMA [26]. This observation is central to matrix-specific interpretation because fecal TMAO is not necessarily the terminal product of a one-way pathway. Microorganisms may use TMAO as an electron acceptor and regenerate TMA, so a low fecal TMAO concentration can coexist with active methylamine metabolism. Conversely, a high concentration may indicate residual availability rather than high absorbed exposure.

The systematic review by Jafari et al. is particularly informative here [15]. Its deliberate separation of circulating, urinary, and fecal outcomes showed both the value and the scarcity of compartment-specific evidence. Only a small fraction of the randomized comparisons reported fecal TMAO; interventions were short; assays and comparators varied; and seafood-containing diets could increase TMAO through direct intake. The review therefore documents cross-matrix heterogeneity but does not validate fecal TMAO as a cardiovascular biomarker. This distinction is precisely why its findings complement rather than duplicate the present conceptual framework.

Analytical reporting is indispensable. A fecal result should state the assay platform, internal standards, calibration, limits of detection and quantification, units, and whether normalization used wet weight, dry weight, total solids, or another denominator. Collection timing, stabilization, storage temperature, freeze-thaw exposure, and stool consistency should also be recorded. Without these details, even two fecal TMAO results may not be directly comparable, and no blood-based hazard estimate can be responsibly transferred to the stool compartment [15,23-26].

Evidence gate 3: absorbed TMA vs. FMO3-dependent TMAO formation

After intestinal absorption, TMA must undergo host biotransformation before it appears as circulating TMAO. Hepatic FMO3 is the principal enzyme involved, but it should not be treated as a fixed conversion constant. FMO3 expression and activity vary with age, sex-related regulation, liver physiology, genetic variation, and potentially interacting dietary or pharmacological exposures [27,28]. The same intestinal TMA delivery can therefore yield different circulating TMAO concentrations in different hosts.

Human data support meaningful interindividual variability in TMA oxidation. Common FMO3 polymorphisms contribute to variation in the TMA-to-TMAO conversion phenotype [28]. In chronic kidney disease, selected variants have been associated with circulating TMAO concentrations, although their relationship with progression and mortality has not been uniform [29]. These observations do not justify genetic determinism, and routine FMO3 genotyping is not yet a standard requirement for every clinical study. They do show that an unmeasured host conversion step can disconnect microbial capacity from the circulating metabolite.

FMO3-related variability complicates both between-person and within-person comparisons. Genetic variants can alter oxidation efficiency, but genotype is only one component of the phenotype; age, sex-related regulation, hepatic function, and the amount and timing of substrate delivery also matter. Consequently, a lower circulating TMAO concentration does not necessarily imply lower intestinal TMA production, and a higher concentration does not identify the microbiota as the dominant source without paired measurements [27-29].

The host-conversion step can also be partly bypassed. Preformed dietary TMAO, particularly from seafood, can enter the circulation without requiring microbial TMA generation or FMO3 oxidation, whereas choline- and carnitine-containing foods depend more strongly on microbial and host conversion. Studies that combine all foods into a single precursor exposure risk attribute biologically different routes to the same mechanism [27-31].

This gate also explains why measuring TMA and TMAO together is more informative than measuring TMAO alone. A high TMA-to-TMAO ratio may be compatible with limited oxidation, but it can also be influenced by timing, intake, absorption, and renal handling. A low ratio may reflect efficient conversion, direct dietary TMAO, or delayed sampling. The ratio is therefore a phenotype to be interpreted, not a stand-alone diagnostic test. Nevertheless, paired TMA and TMAO measurements can reveal discordance that a solitary TMAO concentration conceals [27-31].

Evidence gate 4: circulating concentration vs. production rate

Circulating TMAO is a concentration at a specific sampling time, not a direct measurement of the rate at which the intestinal microbiota produces TMA. It reflects microbial precursor metabolism plus preformed dietary TMAO, intestinal absorption, hepatic formation, tissue distribution, and renal elimination. Fish and seafood can produce rapid, marked plasma and urinary TMAO responses because preformed TMAO is absorbed directly, whereas eggs or meat rely more heavily on precursor metabolism [30]. Chronic red meat feeding can increase systemic TMAO through several mechanisms, including precursor supply, enhanced carnitine conversion, and altered renal excretion [31]. Identical concentrations may therefore arise from different causal histories.

Sampling time can therefore determine the apparent ranking of participants. A postprandial peak after direct dietary TMAO exposure, a delayed response after precursor conversion, and a fasting steady-state value represent different biological questions. Studies should predefine whether they seek acute exposure, pathway responsiveness, or habitual systemic burden and should align blood and urine collection windows with that question [30,31].

Renal function is one of the strongest determinants of circulating TMAO. Concentrations rise as glomerular filtration declines, and elevated TMAO is common in chronic kidney disease [32-34]. Multiomic analyses have likewise identified kidney-related variables among the dominant correlates of plasma TMAO [35]. More recent evidence supports a potentially causal and modifiable relationship between kidney function and circulating TMAO [36]. Thus, impaired clearance can increase the measured concentration even when intestinal production is unchanged.

The kidney-TMAO relationship may also be bidirectional. Experimental work suggests that sustained TMAO exposure can contribute to renal injury, whereas human genetic and longitudinal analyses indicate that reduced kidney function raises TMAO [32,36]. This creates a feedback structure rather than a simple one-directional pathway. In a cardiovascular cohort, a high concentration can therefore reflect increased formation, reduced clearance, disease-related kidney decline, or a combination of these processes [32-36].

Adjustment for estimated glomerular filtration rate (eGFR) is essential but not always sufficient. A single eGFR value may not capture dynamic decline, tubular handling, cystatin-C discordance, or interactions with inflammation and heart failure. Studies should report the renal measure, timing, equation, chronic kidney disease status, and sensitivity analyses with and without renal adjustment. The statement "high circulating TMAO indicates high microbial production" is not justified without challenge, kinetic, or paired-matrix data [32-36].

Longitudinal kidney assessment is especially important in studies with extended follow-up. Baseline renal function may be adequate for prediction modeling but insufficient for causal interpretation if kidney function subsequently declines and simultaneously increases TMAO and cardiovascular risk. Repeated eGFR, cystatin C where available, urinary measurements, and sensitivity analyses excluding advanced kidney disease can clarify whether the observed signal is robust to alternative representations of renal handling [32-36].

Evidence gate 5: prognostic association vs. causal cardiovascular mediation

Even when circulating TMAO is measured accurately and prospectively associated with events, the final transition from biomarker exposure to causal cardiovascular mediation remains distinct. Experimental models provide mechanisms through which TMAO may alter cholesterol transport, vascular inflammatory signaling, platelet calcium responses, and thrombotic potential [1,5,6]. These data establish biological plausibility. They do not automatically establish that the same pathway mediates the observed human hazard ratio at typical concentrations across heterogeneous populations.

MR provides an additional, although not definitive, test of causal direction. A bidirectional MR analysis did not consistently support a direct causal effect of genetically predicted TMAO on coronary artery disease, myocardial infarction, stroke, atrial fibrillation, type 2 diabetes, or chronic kidney disease; instead, genetic liability to type 2 diabetes and chronic kidney disease was associated with higher TMAO, supporting confounding or reverse causation as plausible contributors [37]. Another MR study reported limited signals for TMAO-related metabolites and blood pressure, but the estimates were not uniform across instruments and sensitivity analyses [38].

MR cannot settle the issue by itself. Valid instruments for a metabolite shaped by diet, microbiota, FMO3, and renal clearance are difficult to construct; pleiotropy and weak-instrument bias remain concerns. Nevertheless, the MR literature is sufficient to reject an automatic assumption that the observed direction must run from high TMAO to cardiovascular disease. The evidence is more consistent with a mixture of causal contribution, reverse causation, shared determinants, and population-specific prognostic value [36-38].

Demonstrating mediation requires more than statistical adjustment. Stronger designs would establish temporal order with repeated measurements, quantify both TMA and TMAO, characterize diet and renal function, test an explicit mediator model, and ideally use a selective intervention that changes the pathway and improves a clinically meaningful endpoint. Experimental inhibitors of microbial TMA formation show that the pathway can be modified and can alter thrombosis-related phenotypes [6], but human cardiovascular outcome evidence remains insufficient for routine clinical targeting.

The defensible conclusion is conditional. Circulating TMAO can function as a prognostic biomarker in selected populations and may participate in cardiovascular pathophysiology. It is not a direct readout of stool microbial activity, and neither a single circulating concentration nor a fecal result independently proves that the microbiota mediated an individual cardiovascular outcome [2,7-15,32-43].

Clinical phenotypes and the problem of transportability

Positive prognostic studies span several cardiovascular phenotypes but do not estimate one interchangeable effect. In chronic heart failure, higher circulating TMAO was associated with mortality beyond conventional risk markers [39]. In stable coronary artery disease, a prospective cohort linked higher TMAO with five-year mortality [40]. Among patients presenting with suspected acute coronary syndrome, two independent cohorts showed near- and longer term associations with major adverse events [41]. After ischemic stroke, two cohorts also linked higher TMAO with subsequent cardiovascular events and proinflammatory monocyte profiles [42]. Renal mediation analyses, mechanistic and intervention studies, and analytical validation further illustrate why these associations require cautious interpretation [43-48].

A recent study extended the clinical phenotype to atrial fibrillation. In 5,090 individuals undergoing elective cardiac catheterization, plasma TMAO was independently associated with prevalent atrial fibrillation (adjusted odds ratio 1.7; 95% confidence interval, 1.3-2.1). In complementary mouse models, dietary choline or TMAO accelerated atrial fibrillation, whereas inhibition of microbial CutC/D lowered TMAO and attenuated this phenotype [49]. This combination of human and experimental evidence is mechanistically informative, but the human analysis concerned prevalent rather than incident atrial fibrillation and therefore does not by itself establish prospective cardiovascular mediation.

These studies broaden the clinical signal, but their effect estimates should not be pooled informally or transferred to stool results. Heart failure, stable coronary disease, acute chest pain, stroke, and community prevention cohorts differ in baseline hazard, renal function, medications, inflammation, outcome composition, and competing risks. The same circulating concentration may therefore have different prognostic meaning, and a study-specific quartile cannot be converted into a universal clinical threshold [39-42].

Renal mediation analyses illustrate why transportability and causality require separate testing. In a prospective acute coronary syndrome cohort, the association between TMAO and subsequent cardiovascular events was substantially mediated by eGFR and was no longer statistically significant after additional renal adjustment [43]. This does not make TMAO clinically irrelevant; it shows that the measured concentration may carry prognostic information through kidney-related pathways rather than uniquely through microbial production [43].

Mechanistic evidence and translational limits

Mechanistic experiments provide several biologically plausible routes from systemic TMAO to vascular disease. TMAO exposure has been linked to mitogen-activated protein kinase and nuclear factor kappa B signaling, endothelial inflammatory responses, and NLRP3 inflammasome activation in experimental systems [44,45]. Platelet studies have demonstrated enhanced responsiveness and thrombosis potential [5]. These findings support mechanistic plausibility, but the concentrations, exposure durations, species, cell models, and disease contexts must be compared with those encountered in human observational cohorts before the findings are used as proof of clinical mediation [5,44,45].

Dietary intervention evidence also requires pathway-specific interpretation. Controlled feeding studies show that eggs, red meat, seafood, and other animal-source foods can produce different TMAO responses because they deliver different precursors or preformed TMAO and interact with individual producer phenotypes [30,31,46]. An increase after a defined meal demonstrates exposure responsiveness; it does not by itself establish long-term vascular harm. Conversely, a modest fasting response does not exclude substantial postprandial production or tissue-level effects [30,31,46].

Microbial TMA-lyase inhibition offers a more selective experimental test than broad dietary or antibiotic manipulation. Nonlethal inhibitors reduced TMA/TMAO production and thrombosis-related phenotypes in preclinical models without intentionally eradicating the microbiota [6,47]. This strategy strengthens evidence that the pathway is modifiable, but it remains a translational proof of concept. Human trials demonstrating durable target engagement, safety, and improvement in validated cardiovascular outcomes are still required before TMAO-lowering can be recommended as a therapeutic objective [6,47].

Analytical comparability across biological matrices

Analytical comparability is a prerequisite for biological comparison. Stable-isotope dilution liquid chromatography-tandem mass spectrometry is widely used for plasma TMAO, and nuclear magnetic resonance methods have also been validated for serum and plasma [2,48]. Agreement within one matrix does not guarantee agreement in stool, where extraction recovery, water content, microbial activity, and normalization introduce additional sources of variation. Reports should identify the method and matrix rather than refer generically to a "TMAO level" [15,23-26,48].

Cross-study synthesis should also distinguish concentrations below quantification limits, imputed values, normalized ratios, and absolute measurements. Unit conversion cannot correct for different sample handling or for assays that measure different molecular targets. Ideally, paired-matrix studies should analyze all samples in the same laboratory with prespecified quality controls, report technical coefficients of variation, and include replicate or reference materials appropriate to each matrix [15,25,26,48].

Dietary context and precursor specificity

Dietary exposure should be decomposed into chemically and biologically distinct inputs. Choline, phosphatidylcholine, carnitine, betaine, and preformed TMAO do not enter the pathway at the same point, and their conversion depends on food matrix, dose, habitual intake, and the available microbial functions. Combining them into a single category of "TMAO-producing foods" conceals whether a measured response arose from direct absorption, microbial TMA formation, host oxidation, or altered excretion [3,15,22,30,31,46].

Seafood is a particularly important interpretive challenge because it can deliver preformed TMAO and generate a rapid systemic rise that bypasses the microbial TMA step. Red meat, eggs, and carnitine or choline challenges more directly test precursor conversion but still produce heterogeneous responses among participants. The direction and magnitude of a diet-induced change should therefore be reported together with the specific food, precursor dose, habitual diet, fasting interval, and sampling curve rather than interpreted from the terminal concentration alone [15,22,30,31,46].

Habitual exposure may also alter the response to an acute challenge. Microbial communities can adapt to repeated substrate availability, and producer phenotypes may differ between individuals with contrasting dietary patterns. This makes an acute challenge useful for measuring pathway responsiveness but not a complete substitute for long-term exposure assessment. Combining standardized challenges with repeated free-living measurements offers a more informative view of both capacity and habitual burden [3,22-25,30,31].

A tiered design for stronger causal inference

Study design can be matched explicitly to the intended claim. A matrix-specific cross-sectional study can describe distributions and associations at one time point. A paired-matrix repeated-measures study can test within-person coherence among stool, blood, and urine. A standardized precursor challenge can examine pathway responsiveness, while longitudinal mediation and selective intervention are needed to approach causal cardiovascular inference. Labeling these tiers prevents exploratory studies from being judged by standards they were not designed to meet while preventing causal language from exceeding the design [15,22-38,43].

Statistical analysis should also reflect the measurement structure. Microbial relative-abundance data are compositional, repeated metabolite values are correlated within participants, and values below quantification limits should not be treated as ordinary zeros. Multiple testing, missingness, batch effects, renal covariates, and prespecified interaction terms require transparent handling. Prediction models should separate development from validation and report calibration as well as discrimination [10,15,23-25,35,43,48].

Discordant and null findings should be retained because they identify where the pathway may break. A cohort with microbial capacity but no systemic response, or a systemic response without an outcome association, is not merely a failed replication; it can localize the influence of diet, absorption, host conversion, clearance, or population context. Reporting these patterns alongside positive results is essential for testing the evidence-gated model rather than using it only to explain associations after they appear [10,13-15,23-26,36-38,43].

A matrix-specific conceptual framework for interpretation

The biological matrix is not a technical footnote; it defines the claim that can be made. The five evidence gates are presented as a matrix-specific conceptual framework: a heuristic for organizing observations and identifying the additional evidence needed before moving from one biological level to the next. It is not a validated causal model or scoring instrument, and the order of the gates should not be interpreted as proof that the full sequence operates in every individual.

Passage through gate 1 requires functional evidence of TMA production, such as gene expression, enzyme activity, targeted metabolites, isotope tracing, or a standardized precursor challenge. Gate 2 requires paired fecal and systemic measurements with attention to stool output or normalization, diet, transit, and timing. Gate 3 requires paired TMA and TMAO measurements together with relevant FMO3 context. Gate 4 requires renal measures, repeated sampling, or kinetic data that separate production from clearance. Gate 5 requires temporal evidence, mediation analysis, and ideally a selective intervention linked to a clinical outcome. Table 1 summarizes the evidential ceiling of common measurements and the additional evidence required for the next inference [2,7-15,21-43,48,49].

**Table 1:** A matrix-specific conceptual framework derived from the literature reviewed on TMA/TMAO The table summarizes what each measurement can support, what it cannot establish on its own, and the evidence required before proceeding to the next inference TMA: trimethylamine; TMAO: trimethylamine N-oxide; FMO3: flavin-containing monooxygenase 3 Source: [2,7-15,21-43,48,49]

Measurement	What it can support	What it cannot establish alone	Evidence needed for the next inference
Fecal taxa [21]	Presence or relative abundance of organisms potentially involved in methylamine metabolism	Active TMA production, circulating TMAO, or cardiovascular risk	Functional genes, substrate exposure, activity measurements, and metabolite flux
Microbial genes or predicted pathways [21-25]	Potential biochemical capacity for TMA production or utilization	Gene expression, direction or magnitude of flux, or absorbed dose	Metatranscriptomics, proteomics, targeted metabolites, or challenge testing
Fecal TMA [22-26]	A local luminal TMA signal at the time of sampling	The amount absorbed or converted by the liver	Stool output or normalization, diet and transit data, and paired plasma or urine measurements
Fecal TMAO [15,26]	Matrix-specific intestinal availability and transformation	Plasma TMAO, systemic exposure, or validated cardiovascular risk	Paired biological matrices, assay validation, repeatability, and outcome validation
Plasma or serum TMAO [2,7-14,27-36,48]	Integrated systemic concentration at the time of sampling	Exclusive microbial origin, production rate, or causal mediation	Diet, paired TMA measurement, FMO3 context, renal function, and repeated measurements
Urinary TMAO [15,25,30,31]	Systemic exposure combined with renal excretion during the collection interval	Microbial production independent of renal handling	Timed collection, volume or creatinine normalization, paired plasma measurements, and renal assessment
Association with major adverse cardiovascular events [7-14,37-43,49]	A population-specific prognostic association	Individual causality or a microbiota-mediated mechanism	Temporal replication, mediation analysis, selective intervention, and clinical outcome evidence

Implications for stool-based research and commercial reports

Commercial stool reports increasingly include taxonomic, functional, and metabolite outputs. These data can be useful for hypothesis generation, but the matrix and method must remain visible in the language of the analysis. A result labeled "TMAO" in a stool report should not be renamed "plasma TMAO," "circulating TMAO," or "systemic TMAO exposure" [15,21-26]. Previous studies [7-15,39-43] support population-specific prognostic associations for circulating TMAO, but they do not validate a clinical threshold for fecal TMAO. Clinical cutoffs derived from blood cohorts should therefore not be transferred to stool measurements. A fecal threshold would require paired-matrix studies demonstrating reproducible correspondence with circulating exposure and independent outcome validity.

For report-derived research datasets, a defensible term is "TMAO reported in the fecal metabolite section of the commercial assay." Associations with body mass index, visceral adiposity, lipids, glucose, blood pressure, or cardiovascular diagnoses should be described as exploratory associations between a fecal report-level measurement and cardiometabolic features. A positive association does not validate a systemic biomarker; a null association does not contradict the plasma literature [15,23-26].

The same discipline applies to microbial genes and taxonomy. A study can report enrichment of potential TMA-producing pathways, but the conclusion should remain at the level measured unless paired metabolites demonstrate downstream coherence. Ideally, cardiovascular microbiome cohorts should analyze taxonomy, functional potential, fecal metabolites, host biochemistry, renal function, and clinical outcomes as distinct but linked domains. Concordance across levels is evidence; substitution of one level for another is not [21-25].

An efficient prospective design would collect a standardized dietary history; fasting plasma or serum TMA, TMAO, choline, carnitine, and betaine; timed or normalized urinary TMAO; a stool sample analyzed by a validated targeted method; functional characterization of TMA pathways; repeated kidney-function measures; and relevant cardiovascular phenotyping. A standardized precursor challenge may be more informative than a single fasting value because it tests pathway responsiveness [15,22-38,43,48].

Minimum reporting standards

Table 2 translates the inferential framework into a minimum reporting set. The purpose is not to make every study maximal but to prevent avoidable ambiguity about what was measured and which transition remains untested [15,23-38,43,48].

**Table 2:** Minimum reporting elements for interpretable TMA and TMAO studies References shown within the table identify key supporting evidence for each reporting domain CKD: chronic kidney disease; eGFR: estimated glomerular filtration rate; FMO3: flavin-containing monooxygenase 3; LOD: limit of detection; LOQ: limit of quantification; TMA: trimethylamine; TMAO: trimethylamine N-oxide Source: [15,23-38,43,48]

Domain	Minimum information	Why it matters	Common bias if omitted
Biological matrix [15,23-26]	Specify plasma, serum, urine, wet stool, dry stool, or report-derived output	Defines the biological process represented by the measurement	Cross-matrix extrapolation
Analytical method [15,23-26]	Report the platform, internal standards, calibration, LOD/LOQ, units, and normalization method	Determines analytical validity and comparability	Assay mismatch or scale mismatch
Diet and timing [22-24,30,31]	Report fasting status, recent fish, red meat, and egg intake, habitual diet, and collection interval	Helps distinguish direct dietary exposure from precursor metabolism and transient responses	Dietary misattribution
Microbial function [21-25]	Report functional genes together with expression, activity, targeted metabolites, or challenge-derived producer phenotype when feasible	Distinguishes potential biochemical capacity from realized metabolic flux	Functional overinterpretation
Fecal context [15,23-26]	Report stool consistency, transit, stabilization, storage, wet- or dry-weight normalization, and repeatability	Addresses dilution, degradation, and within-person variability	Residual-matrix bias
Host conversion [27-29]	Report paired TMA, liver status, and FMO3-relevant phenotype or genotype when justified	Accounts for interindividual variation in hepatic oxidation	Host conversion attributed to the microbiota
Renal handling [29,32-36]	Report eGFR method, creatinine or cystatin C, CKD status, urinary normalization, and timing	Helps separate metabolite formation from impaired clearance	Clearance attributed to production
Outcome inference [7-14,37,38]	Report temporal sequence, confounder control, mediation analysis, intervention, and clinical endpoint definition	Aligns causal language with the study design	Association reported as mechanism

Limitations of this review

This article is a critical narrative review informed by a structured search, not a systematic review. The search was not prospectively registered, the number of retrieved records was not logged in PRISMA format, screening was not duplicated, and no formal risk-of-bias instrument or evidence-grading system was applied. The direct fecal TMAO literature remains sparse and analytically heterogeneous; several conclusions therefore rely on biologically adjacent evidence rather than large paired-matrix cardiovascular cohorts. Cardiovascular studies differ in fasting status, sample timing, assay methodology, renal adjustment, and endpoint definitions. FMO3-related variation is incompletely characterized in general populations, and MR analyses depend on instrument validity and may not capture environmentally determined metabolite exposure. Finally, the proposed gates are a conceptual interpretive framework rather than a validated causal model or scoring instrument. These limitations reinforce the central argument: claims about one matrix or biological level require evidence generated at that level and should not be imported from an adjacent compartment [10,15,23-38,43,48,49].

## Conclusions

Circulating TMAO has reproducible prognostic associations in several cardiovascular populations, but microbial potential, luminal TMA flux, fecal TMA/TMAO, systemic exposure, and clinical outcomes remain distinct biological levels. No single fecal, plasma, or urinary measurement identifies production, absorption, host oxidation, renal clearance, and causal mediation simultaneously. Each inferential transition therefore requires matrix-matched evidence and explicit control of diet, sampling, FMO3-related host variability, and kidney function.

Current evidence does not yet support fecal TMA or TMAO as validated surrogates for circulating exposure or as independently validated cardiovascular risk markers. This does not exclude a future clinical role for stool-based measurements; rather, it defines the validation that would be required. The most informative studies will pair biological matrices, quantify TMA and TMAO, standardize exposure and timing, repeat renal and systemic measurements, and test temporality, mediation, and selective intervention before making causal cardiovascular claims.
